# Multidimensional evaluation and strategic framework for integrated development of urban agglomerations in underdeveloped regions

**DOI:** 10.1371/journal.pone.0345887

**Published:** 2026-04-02

**Authors:** Fang Wang, Xin Li

**Affiliations:** 1 School of Environment and Urban Construction, Lanzhou City University, Lanzhou, China; 2 College of Geography and Environmental Science, Northwest Normal University, Lanzhou China; Zhejiang A and F University, CHINA

## Abstract

As a complex spatial giant system leading regional development, studying the integration level and coupling drive of urban agglomerations is of great significance for the sustainable development of urban agglomeration systems. This study established an integrated evaluation index system for the Lanzhou-Xining urban agglomeration (LXUA) and analyzed the spatio-temporal evolution characteristics of multidimensional and comprehensive integration. In addition, based on the parameter optimal geographic detector model, the coupling driving mechanism of the interaction between variable factors on the comprehensive integration of urban agglomerations was revealed. Research has found that: (1) In terms of temporal changes, there are significant differences in the integration level of different dimensions in the LXUA. Among them, the integrated indices of economic development, public services, and urban space have relatively small fluctuations, while the integrated indices of ecological environment and infrastructure have relatively large fluctuations. The problem of insufficient and unbalanced regional economic development in the LXUA is particularly prominent. (2) In terms of spatial distribution, the spatial differentiation characteristics of the integration level of each dimension are obvious, with high-value areas mainly concentrated in core cities, while underdeveloped counties lag behind in development. The over-all integration level of the LXUA has been improved, but there is a prominent imbalance between dimensions and regions. It is necessary to strengthen cross regional cooperation and policy tilt to address the shortcomings of the economy and infrastructure, in order to achieve sustainable development. (3) The independent explanatory power of economic development and infrastructure factors on the comprehensive integration of urban agglomerations is strong through factor interaction detection. The interaction between infrastructure, public services, and urban spatial fac-tors has a nonlinear enhancement effect on the comprehensive integration of urban agglomerations. The research conclusion is helpful for formulating sustainable development strategies for urban agglomerations.

## 1. Introduction

With the acceleration of global urbanization, urban agglomerations have become the core carrier of regional economic development and the main spatial form of population agglomeration. According to United Nations data, by 2030, 60% of the global population will reside in urban agglomerations, which account for over 75% of global resource consumption [1]. However, the traditional extensive urbanization model has led to increasingly prominent problems such as resource overload, ecological degradation, and social differentiation [2]. It is urgent to conduct systematic research to evaluate the development level of urban agglomerations and further explore new paths for sustainable development of urban agglomerations.

As early as the late 19th century, research on regional integration development had already received attention in developed Western countries. After more than a hundred years of development, numerous achievements have been made in theoretical foundations and empirical research [3,4]. As a complex spatial aggregation system leading regional development, urban agglomerations have become a consensus in academia. The current research on urban agglomerations mainly focuses on six aspects: spatial layout and structure of urban agglomerations [5,6], regional economic integration [7, 8], transportation and infrastructure construction [9], ecological environment and sustainable development [10, 11], social governance and public services [12,13], policy coordination and institutional innovation [14, 15], etc. The research on the integration of urban agglomerations is also becoming increasingly rich. There are currently two main methods for measuring urban integration: one is to select a single indicator based on different perspectives for measurement, such as economic integration, industrial integration, population integration [16, 17], market integration [18], resource integration [19], spatial integration [20], etc. Another approach is to establish a comprehensive evaluation index system to measure the level of integration of urban agglomerations from multiple perspectives. Li et al. constructed a comprehensive evaluation index system from three dimensions—economic, social, and environmental—which covers core dimensions of urban agglomeration integration such as economic coordination and joint environmental governance. The results revealed deficiencies in the spatial linkage development of China’s three major urban agglomerations, namely the Pearl River Delta, the Yangtze River Delta, and the Beijing-Tianjin-Hebei urban agglomerations [21]. Lu et al. took metropolitan areas as the research object and established a comprehensive evaluation system from two aspects: development status and development potential. This provides a reference for the refined assessment of key dimensions of urban agglomeration integration, and is particularly significant for studying the integration differences between urban hierarchical levels within urban agglomerations [22].

Overall, a single indicator measurement can clearly reflect the specific development characteristics of urban agglomerations, facilitating in-depth analysis of specific issues. However, measuring from a single dimension alone cannot fully reflect the complexity and multidimensional characteristics of urban ag-glomeration system integration. Comprehensive indicator measurement can compensate for this. Integrating evaluation indicators from multiple dimensions can not only comprehensively reflect the overall level of urban agglomeration integration, but also comprehensively consider the interaction and influence between various factors, which is more in line with the actual situation of urban agglomeration integration development.

International scholars tend to focus more on cross-border regions within Europe or Asia when studying the process of regional integration. Dutch scholar Meijers et al. [23] and American scholar Aggarwal et al. [24] respectively conducted research on the level of economic integration in 117 urban agglomerations in Europe and Northeast Asia. Chinese scholars focus on the research of regional integration of urban agglomerations, mainly selecting the overall urban agglomerations such as the Yangtze River Midstream urban agglomeration, Beijing-Tianjin-Hebei urban agglomeration, Yangtze River Delta urban agglomeration, Chengdu-Chongqing urban agglomeration, Changsha-Zhuzhou-Xiangtan urban agglomeration, etc. Some scholars also pay attention to typical regions within urban agglomerations. But overall, scholars tend to choose regions with more developed development and pay less attention to underdeveloped areas. Cities in underdeveloped areas often have relatively weak economic strength, technological innovation, social ser-vices, and significant differences in development between cities, leading to insufficient economic synergy within urban agglomerations [25].

The Lanzhou-Xining urban agglomeration (LXUA) is a key inter-provincial urban agglomeration in western China. Its planned scope transitions from the Loess Plateau to the Qinghai-Tibet Plateau, strategically located at a critical node of the “Belt and Road” Initiative. From the perspective of ecological security, situated in the upper reaches of the Yellow River, the LXUA serves as an important water conservation and recharge area, as well as a major ecological barrier in the Yellow River Basin. It plays a unique strategic role in preventing the eastward spread of desertified areas in western China and fulfills essential service functions for restricted and prohibited development zones. In terms of optimizing the national territorial development pattern, this region possesses favorable development conditions and substantial potential in northwest China. Fostering the development of the urban agglomeration is conducive to consolidating border defense in western China, safeguarding national territorial security, and promoting ethnic exchange, interaction, and integration. With the construction of the New Western Land-Sea Corridor and the connection to the comprehensive transportation corridors linking Sichuan, Chongqing, Yunnan, Guizhou, and Guangxi, the comprehensive hub functions of Lanzhou and Xining have been strengthened, making the LXUA a crucial pivot for advancing China’s opening-up to the west and south. In recent years, the accelerated construction of bases for new energy, new materials, and circular economy in the LXUA has strongly supported the development of northwest China, emerging as an important economic growth pole.

To further explore the level of regional integration of the LXUA more explicitly, this study constructs a comprehensive evaluation index system for its integration from five dimensions: economic development, ecological environment, infrastructure, public services, and urban space. The research aims to investigate the level of regional integration and analyze the sustainable development capacity of the LXUA. By exploring the impact of single-factor and dual-factor interaction effects on the comprehensive integration of the urban agglomeration, the coupled driving mechanism of its comprehensive integration is revealed. Ultimately, this study seeks to contribute to the regional integration and sustainable development of urban agglomerations in underdeveloped regions.

## 2. Materials and methods

### 2.1. Study area

The LXUA (99 ° 01 ‘-105 ° 38’E, 34 ° 51’ −37 ° 38’N) is located in the upper reaches of the Yellow River in western China, with high terrain in the west and low terrain in the east. Mainly characterized by temperate continental climate, with significant regional differences. As one of the 19 key urban agglomerations under construction in China, the LXUA spans across Gansu and Qinghai provinces, with the two provincial capitals (Lanzhou and Xining) as its core. The specific scope includes Lanzhou, Baiyin, Dingxi, and 39 counties (districts) in Gansu Province, as well as Xining and Haidong in Qinghai Province, with a total area of 9.75 × 10^4^km^2^. As of 2020, the total population of 39 counties (districts) in the LXUA was 134,600, with a population density of 125 people/km^2^. The regional GDP was 613,900 yuan, the overall GDP was 664.8 billion yuan, and the per capita GDP was 51,139 yuan. The LXUA is not only the core area of national ecological security barrier construction, but also a key area for poverty alleviation in the mountainous areas of the Qinghai Tibet Plateau in northwest China. Therefore, measuring and analyzing the integration level of the LXUA, identifying the current development shortcomings, is conducive to further stimulating the development momentum of the urban agglomeration and promoting high quality regional economic, social, and ecological development.

### 2.2. Construction of evaluation index system for urban agglomeration integration

#### 2.2.1. Principles for selecting indicators.

**Fig 1:**
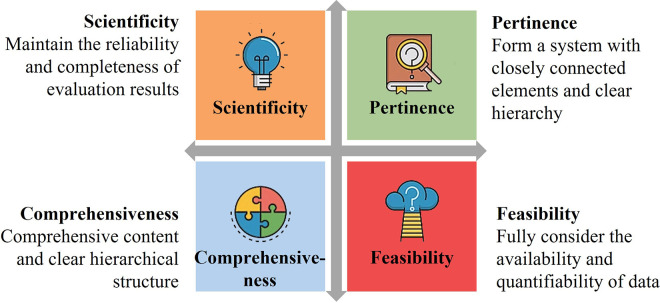
Principles and framework for selecting evaluation indicators.

In February 2018, the Chinese government issued the “Lanzhou Xining Urban Agglomeration Development Plan”, which clearly stated the need to build a spatial pattern that is compatible with the carrying capacity of resources and environment, promote eco-logical co construction and environmental governance, create a green circular industrial system, promote infrastructure interconnection, comprehensively enhance the level of openness and cooperation, and establish a sound mechanism for coordinated development [26]. A scientifically reasonable indicator system is the core content of evaluating the integration level of urban agglomerations, and plays an important role in obtaining reliable evaluation results. This study mainly adheres to four basic principles when constructing the indicator system: scientificity, comprehensiveness, pertinence, and feasibility (as shown in Fig 1).

#### 2.2.2. The process of selecting indicators.

Based on the principles of indicator selection and the actual development of the LXUA integration, and drawing on relevant research on measuring regional integration levels by domestic and foreign scholars [9,27,28], a comprehensive measurement index system for the LXUA integration was constructed from five dimensions: economic development, ecological environment, infrastructure, public services, and urban space. The research aims to evaluate the level of integration within the LXUA from the perspectives of single dimensional integration, regional integration, and comprehensive integration.

**Integration of economic development**: The integration of economic development aims to promote the optimization and co-ordination of the internal economic structure of urban agglomerations, enhance the over-all economic competitiveness and sustainable development capacity of urban agglomerations [29, 30]. This study constructs an integrated evaluation index system for economic development from three dimensions: economic development quality, financial market activity, and fiscal expenditure share.

**Integration of ecological environment**: With the rapid development of urban agglomeration economy and society, the eco-logical environment is facing great challenges, which goes against the development law of economic development and environmental protection walking in the same direction. The integration of ecological environment in urban agglomerations can achieve the sharing and allocation of resources within urban agglomerations, such as water resources, land resources, energy, etc., reducing resource waste and over-development, and improving resource utilization efficiency. At the same time, we can also jointly address ecological and environmental issues such as air pollution, water pollution, soil pollution, etc., protect and improve the overall ecological environment of urban agglomerations, and enhance the stability and anti-interference ability of ecosystems [31]. The study constructed integrated evaluation indicators for the ecological environment of urban agglomerations from three aspects: the level of public green space construction, atmospheric ecological protection, and water ecological protection.

**Integration of infrastructure**: Infrastructure integration refers to the unified planning, construction, and management of infrastructure between different cities within a city cluster, achieving inter-connectivity and coordinated development of infrastructure. Infrastructure integration includes infrastructure construction in areas such as transportation, energy, water conservancy, communication, and information technology [32]. The study constructed integrated evaluation indicators for the ecological environment of urban agglomerations from three aspects: highway construction level, postal and telecommunications level, and public education construction level.

**Integration of public services**: Integration of public services refers to the integration of public service resources, optimization of public service layout, and improvement of public service levels among different cities within the scope of urban agglomerations, achieving the sharing and inter-connection of public services, and providing higher quality and more convenient public services for residents of urban agglomerations [33]. The integration of public services aims to improve the overall level of public services in urban agglomerations, promote collaborative development and social progress within urban agglomerations. Research on constructing an integrated evaluation index system for public services in the LXUA from three aspects: education services, medical services, and social security services.

**Integration of urban space**: Urban spatial integration refers to the integration of urban spatial resources, optimization of urban spatial layout, strengthening of connections and interactions between different towns within urban agglomerations, achieving coordinated development and organic connectivity of urban spaces. Urban spatial integration aims to improve the overall urban planning and development level of urban agglomerations, promote coordinated urban development and resource sharing within urban agglomerations [34]. Research on constructing an evaluation index system for urban spatial integration in the LXUA from three aspects: urbanization rate, urban-rural integration, and urban-rural income gap.

In conclusion, the comprehensive evaluation index system constructed in this study is shown in Table 1.

**Table 1:** Evaluation index system for urban agglomeration integration.

First level indicator	Secondary indicators	Indicator content	Attribute
Integration of economic development	Economic development quality	Ratio of added value of secondary and tertiary industries to added value of primary industry (%)	+
	Share of fiscal expenditure	Proportion of fiscal expenditure to GDP (%)	+
	Activity level of financial markets	Per capita financial loan balance (10000 yuan)	+
Integration of ecological environment	Construction level of public green spaces	Per capita public green space area (m^2^)	+
	Atmospheric ecological protection	Industrial sulfur dioxide emissions (tons)	–
	Water ecological protection	Urban domestic sewage treatment rate (%)	+
Integration of infrastructure	Highway construction level	Mileage of highways within the administrative region (km)	+
	Postal and telecommunications level	Total volume of postal and telecommunications services (single)	+
	Public education level	Proportion of education expenditure to total investment (%)	+
Integration of public services	Educational services	Primary school teacher-student ratio (%)	–
	Medical service	Number of medical beds per 10000 people (sheets)	+
	Social security services	Number of fixed telephones per 10000 people (sets)	+
Integration of urban space	Urban rural integration	Ratio of rural employees to urban employees (%)	+
	Urbanization rate	Proportion of urban population (%)	+
	Urban-rural income gap	Ratio of per capita disposable income of urban residents to per capita net income of rural residents (%)	+

### 2.3. Data sources and processing

In 2012, the LXUA was still in its initial development phase. Since March 2018, when the Chinese government formally issued the LXUA Development Plan, the agglomeration’s development has followed a structured trajectory. Consequently, this study collected data spanning 2012–2022. Data categories include economic development, ecological environment protection, infrastructure construction, public services, and urban development. To ensure accuracy and scientific validity, all data were sourced from the China County Statistical Yearbook, China City Statistical Yearbook, Gansu Development Yearbook, Qinghai Statistical Yearbook, as well as municipal statistical yearbooks and bulletins from 2013–2022. All datasets maintain consistent statistical standards. The provincial administrative boundaries of China and the county-level administrative boundaries of the LXUA utilised in this study were sourced from the 2022 data of the China Resource and Environmental Science Data Registration and Publication System. Detailed information regarding the data can be accessed at https://www.resdc.cn/DOI/DOI.aspx?DOIID=122. The DEM data originates from the provincial-level DEM 30m data (SRTM 30m) within the Resource and Environmental Science Data Registration and Publication System. Detailed information regarding this dataset can be accessed at https://www.resdc.cn/data.aspx?DATAID=217. All aforementioned datasets are publicly accessible and available for download.

### 2.4. Construction of urban agglomeration integration index

#### 2.4.1. Data standardization processing.

Standardizing raw data effectively eliminates differences caused by varying measurement units, scales, and directional orientations among indicators. Following established methodologies [35], this study applies range standardization (min-max normalization) to preprocess raw data.

Assume there are m urban agglomerations and n evaluation indicators. For positive indicators:


$$\alpha_{ij}=\frac{x_{ij}-min\left\{x_{ij}\right\}}{max\left\{x_{ij}\right\}-min\left\{x_{ij}\right\}},\left(i=1,2,\text{\textbackslash{}ldots},n;j=1,2,\text{\textbackslash{}ldots},m\right)$$

For negative indicators:


$$\alpha_{ij}=\frac{max\left\{x_{ij}\right\}-x_{ij}}{max\left\{x_{ij}\right\}-min\left\{x_{ij}\right\}},\left(i=1,2,\text{\textbackslash{}ldots},n;j=1,2,\text{\textbackslash{}ldots},m\right)$$

where *X*^ij^ denotes the original value of indicator *j* in urban agglomeration *i*, $max\left\{x_{ij}\right\}$ and $min\left\{x_{ij}\right\}$ represent the minimum and maximum values of indicator *j* across all agglomerations.

#### 2.4.2. Calculation of indicator weights.

This study utilizes the coefficient of variation method for comprehensive evaluation. The coefficient of variation method is particularly suitable for cross-regional comparisons at a given time point. It not only assigns weights to individual indicators within the evaluation system but also mitigates the strong subjectivity inherent in pairwise comparison scoring of indicators under subjective weighting methods [36, 37]. The calculation formulas are as follows:


$$S_{j}=\frac{\sqrt{\sum_{j=1}^{n}(x_{ij}-y_{j}{)}^{\text{2}}/n}}{y_{j}}$$

$$W_{j}=S_{j}/\sum_{j=1}^{m}S_{j}$$

Where, *x*_*ij*_ represents the value of region *I* for the *j*-th indicator, *y*_*j*_ is the mean value of the characteristic values for the *j*-th indicator, *n* is the number of regions, and *m* is the number of measurement indicators.

**Fig 2:**
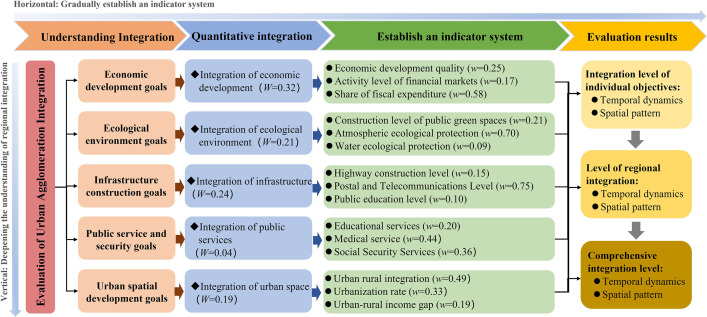
Evaluation indicator system for urban agglomeration integration.

The weight *W*_*j*_ of the comprehensive evaluation index is shown in Fig 2.

#### 2.4.3. Urban agglomeration integration index.

The study employs the linear weighting method to calculate the level of urban agglomeration integration. By standardizing each indicator and then weighting and summing them with their corresponding weights, the integration index for each dimension is obtained.

Regional integration index:


$$I_{r}=\sum_{j=1}^{n}W_{j}\cdot Q_{rj}$$

Where *I*_*r*_ represents the comprehensive integration index for region *r*, and *Q*_*rj*_ represents the standardized and quantized value of the *j*-th indicator for region *r*.


$$I=\sum_{j=1}^{n}W_{j}\cdot Q_{tj}$$

Where *I*_*r*_ represents the comprehensive integration index for the period *t*, and *Q*_*tj*_ represents the standardized and quantized value of the *j*-th indicator for the period *t*.

### 2.5. The optimal parameters geographical detector

The optimal parameters geographical detector is a new practical tool specifically designed to explore the spatial heterogeneity of geographic spatial feature distribution. Firstly, compared to traditional geographic detector models, the new model introduces objective methods for determining the discretization of driving factors, mainly including five grading methods: equal interval, natural breakpoint method, quantile, set interval, and standard deviation. Secondly, by calculating the *q*-value of each continuous factor under different grading methods and different numbers of discontinuities. The *q* value is in the range of 0–1, and the larger the value, the stronger the spatial differentiation of the expansion speed, and the stronger the explanatory power of the influencing factor on its spatial differentiation. Conversely, the smaller the value, the weaker the explanatory power. This study utilized *R* language and implemented model calculations by installing the *GD* program package. The principle and formula of the optimal geographic detector in the parameters mainly refer to existing research [37,38].

## 3. Results

### 3.1. Time series changes in the integration level of LXUA

#### 3.1.1. Characteristics of changes in integration levels across different dimensions.

**Fig 3:**
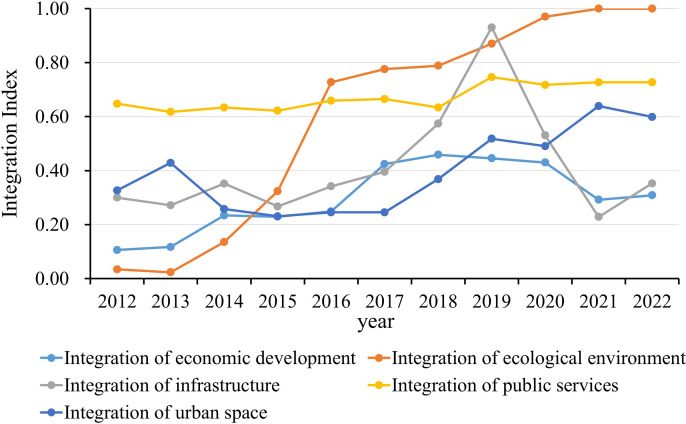
Time series evolution of integration level of LXUA.

There are significant differences in the integration level of different dimensions in the LXUA (as shown in Fig 3). During the research period, the economic development integration index, public service integration index, opening-up integration index, and urban spatial integration index all had relatively small fluctuations, while the ecological environment integration index and infrastructure integration index had larger fluctuations. From the average level of integration in various dimensions from 2012 to 2022, the level of integration in public services is the highest, followed by integration in ecological environment, while the level of integration in economic development is the lowest. This indicates that the problem of insufficient and unbalanced regional economic development in the LXUA is more prominent.

Specifically, the overall economic integration development of the LXUA shows a fluctuating upward trend, but the overall development level is relatively low. The increase in economic fluctuations may indicate that the LXUA has experienced relatively stable economic growth during this period and has a certain degree of momentum. This is related to the performance of enterprise investment, consumption expenditure, exports, and other aspects within the urban agglomeration. For example, in terms of corporate investment, Lanzhou, Xining and their surrounding areas have launched resource-complementary industrial cooperation based on their unique industrial resources and industrial foundations. Qinghai Salt Lake Industry Co., Ltd. has collaborated with Lanzhou Petrochemical Company to develop lithium battery materials. By leveraging Lanzhou’s chemical technology advantages and Qinghai’s abundant salt lake resource advantages, the development of the lithium battery materials industry has been promoted, making it a typical case of resource complementarity-driven industrial integration. At the same time, different cities in the LXUA may have complementarity in terms of economic structure, industrial layout, human resources, etc. Through cooperation and interaction, resource optimization and collaborative development effects can be achieved, thereby promoting the overall economic level improvement. In addition, the government may provide a series of preferential policies for urban agglomerations, such as tax reductions, subsidies, priority development projects, etc., to attract and support the development of enterprises, thereby promoting the economic integration of the entire urban agglomeration.

The overall level of ecological environment integration is on the rise. The level of ecological environment integration was extremely low from 2012 to 2013, with a slight decline; The level of ecological environment integration has significantly increased from 0.0235 to 0.7275 from 2013 to 2016; The growth trend has slowed down from 2016 to 2022, and the integration index is approaching 1. This indicates that in the early stages of urban agglomeration development, ecological environment protection and governance faced severe challenges, and there was insufficient coordination and cooperation between regions. The LXUA is located in a key water conservation area and a priority soil and water conservation zone in the upper reaches of the Yellow River. With the organization and implementation of the “Special Plan for Ecological Protection and High-Quality Development of the Yellow River Basin”, the regional ecological environment has been effectively improved, and the stability of the regional ecosystem has been significantly enhanced. The current level of regional ecological environment integration has reached a relatively high level. In the future, the focus of ecological environment integration development should shift towards finer details and take deeper measures to further enhance it.

The level of infrastructure integration in the LXUA showed a trend of first increasing and then decreasing in 2021−2022, reaching its highest value of 0.9303 in 2019. The lowest level of infrastructure integration occurred in 2021, with a minimum value of 0.2287. Affected by the COVID-19, after 2019, the infrastructure integration level of LXUA showed a downward trend, which seriously hindered the integration process of urban agglomeration, and formed a “trough period” in 2021.

The integration level of public service development is showing a fluctuating upward trend, and it is relatively slow. At the overall level of the LXUA, the integration index of public service facilities has increased from 0.64 to 0.72, with an average annual growth rate of 1.01%, showing a significant upward trend and a significant improvement in the level of public service integration. From the perspective of changing trends, the phased characteristics of public service integration in the LXUA are obvious. From 2012 to 2018, the level of public service integration was around 0.65, and since 2018, the index of public service integration has risen from 0.65 to above 0.7. This improvement is closely intertwined with the goal of the “14th Five-Year Plan for Western Development” (FYPWD) focusing on regional coordination and infrastructure upgrading in western China. The plan has not only promoted the construction of a multi-level transportation network in the LXUA but also accelerated the development of regional power grid projects and supporting water conservancy facilities, such as the West Route of China’s South-to-North Water Diversion Project.

The overall level of urban spatial integration in the LXUA showed a fluctuating upward trend from 0.3265 to 0.5990 from 2012 to 2022, with an average annual growth rate of 83%. Among them, the level of urban spatial integration remained relatively stable from 2014 to 2017, while the period from 2017 to 2019 was the fastest growing time for the level of urban spatial integration in the LXUA. Specifically, there were relatively small differences in urban spatial integration among different regions in 2013. After 2013, except for Weiyuan County in Dingxi City, the rest of the regions had a flat change from 2014 to 2017, and showed a fluctuating upward trend from 2019 to 2022.

#### 3.1.2. Characteristics of changes in the level of comprehensive integration.

**Fig 4:**
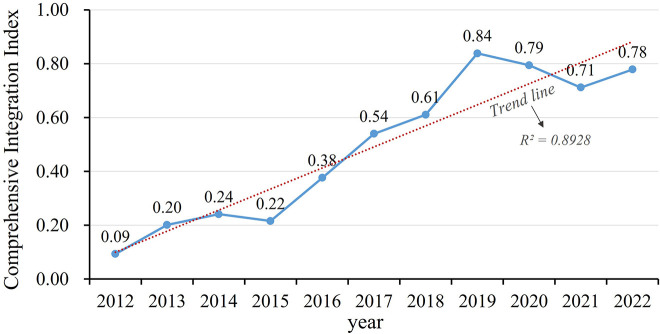
Comprehensive integration level of LXUA from 2012 to 2022.

Calculate the comprehensive integration index of the LXUA, and the overall trend is shown in Fig 4. From 2012 to 2022, the overall level of comprehensive integration in the LXUA showed an upward trend. In terms of temporal changes, it presents phased development characteristics: 2012–2015 is the initial development stage, 2015–2019 is the accelerated development stage, 2019–2021 is the slow development stage, and 2021 to present is the recovery development stage.

From 2012 to 2015, the LXUA was in the initial stage of development, with a gradual upward trend in integration level. During this period, urban agglomerations may be in the initial stage of integration, and various infrastructure and cooperation mechanisms are still being established and improved, so the improvement of integration level is relatively slow. From 2015 to 2019, urban agglomerations entered an accelerated development stage, especially after the release of the “Lanzhou Xining Urban Agglomeration Development Plan” in 2018, the level of integration improved rapidly in 2018–2019. The introduction of the plan provides clear direction and policy support for the integrated development of urban agglomerations, promoting rapid improvement in the level of integration. At the same time, this period may also be accompanied by the interconnection of transportation networks, further narrowing the time and space distance between Lanzhou and Xining, as well as the joint formulation and implementation of the “Lanzhou Xining Urban Agglomeration Development Plan” by the two provinces, achieving cross provincial medical insurance mutual recognition and other measures, promoting the comprehensive improvement of the regional ecological environment governance system and governance capacity. From 2019 to 2021, due to the impact of the international epidemic, development in various aspects has been slow, and the level of integration has decreased. The epidemic has had a serious impact on economic activities, personnel mobility, and social interaction, hindering the integration process. Since 2021, after taking a series of recovery measures, the economic development of the LXUA has recovered and integrated development has shown a good momentum. This indicates that while urban agglomerations are responding to the challenges of the epidemic, they are also actively taking measures to promote economic and social recovery, and integrated development is returning to an upward trajectory. In addition, the Lanxi urban agglomeration is gradually moving towards integrated development, and the synergistic effect of ecological civilization construction is becoming increasingly prominent.

### 3.2. The spatial pattern evolution of LXUA

#### 3.2.1. The spatial pattern of integrated economic development.

From the perspective of the spatial pattern of comprehensive economic development, the regions with high levels of integration are Lanzhou City, Baiyin District, Pingchuan District, and Xining City, with average economic development integration indices of 0.6963, 0.5191, 0.4834, and 0.4628, respectively. These regions have a relatively complete secondary and tertiary industry system, providing strong support for the integration of urban agglomeration economic development. The abundant mineral resources and land resources provide important material basis for the integrated economic development of urban agglomerations. At the same time, these regions have a high level of regional cooperation and close economic ties, which have promoted the integration of urban agglomeration economic development. The areas with lower levels of economic integration are Jingyuan County and Yongtai County, with average economic integration indices of 0.0719 and 0.0945, respectively. The development level of the secondary and tertiary industries in these regions is relatively low, making it difficult to meet the needs of integrated economic development in urban agglomerations. At the same time, due to relatively backward transportation infrastructure and insufficient connection with regions with higher levels of economic development, the speed of integrating into the economic development of urban agglomerations is slow.

#### 3.2.2. The spatial pattern of integrated ecological environment.

From the spatial pattern of ecological environment integration in various regions of LXUA, Guinan County has the highest level of ecological environment integration, while Lanzhou City has the lowest level of ecological environment integration. The main reason is that the government of Guinan County actively increases its efforts to control environmental pollution, closely relies on national ecological construction projects and promotes the three-year action plan for national land greening, and continues to implement a number of ecological key projects such as comprehensive management of the ecological environment around the south bank of the Longyangxia reservoir area, subsidies for the Tianbao public welfare forest, and ecological protection and construction of the Three Rivers Source. This further strengthens the green foundation of Guinan, effectively solves local environmental problems, improves the ecological environment, and enhances the level of ecological environment integration. As the provincial capital city, Lanzhou has a fast urbanization process and rapid population growth. Urban expansion has encroached on ecological land and caused certain pressure on the ecological environment. At the same time, the industrial structure of Lanzhou City is dominated by heavy industry, with a large proportion of traditional industries. High energy consuming and high emission industrial enterprises dominate the city’s economic development. This industrial structure has led to serious ecological and environmental pollution problems in Lanzhou City, and the level of ecological and environmental integration is relatively low.

#### 3.2.3. The spatial pattern of integrated infrastructure.

From the spatial pattern of comprehensive infrastructure level in urban agglomerations, the level of infrastructure integration in Lanzhou City is much higher than in other regions, with the highest integration value occurring in 2015 at 0.6396; Next is Xining City, with the highest level of integration in 2019, with a value of 0.6262. The area with the lowest level of integration is Lintao County in Dingxi City. From 2012 to 2022, the average value of infrastructure integration was 0.3018. Far below the average level of integration within the region. Lanzhou and Xining, as the two core points in the integration process of the LXUA, have a relatively large population base, which makes their integration level in the entire region not outstanding. On the contrary, some areas with smaller population bases but better economic development, such as Baiyin District, Pingchuan District, and Anding District, have relatively stable levels of integration during the research period. In addition, the development of Lanzhou, Xining and other large cities after 2019 reflects that the COVID-19 has a greater impact on the integration level of large cities than small counties.

#### 3.2.4. The spatial pattern of integrated infrastructure.

From 2012 to 2020, the integrated development index of public services in the LXUA was between 0.005–0.961, mainly concentrated around 0.2. Xining City has a relatively high level of integration, followed by Lanzhou City, and Haidong City has the lowest level. The integration level between Baiyin District and Pingchuan District is significantly higher than that of other cities. The average integration index from 2012 to 2020 was above 0.6. Relatively speaking, the integration level of Dongxiang Autonomous County is relatively low, with indices not exceeding 0.1. Due to the varying degrees of emphasis on basic public services in urban agglomerations, resource allocation is more likely to be in the economic field, and there is insufficient attention and emphasis on the development of basic public services. The development of basic public services is unbalanced between urban and rural areas, as well as between regions in some cities

#### 3.2.5. The spatial pattern of integrated urban space.

From the perspective of the spatial pattern of urban spatial integration, the high value areas are concentrated around Lanzhou city, while low value areas are concentrated on the west side of the LXUA. The highest level of urban spatial integration in 2012 was in Pingchuan District, Baiyin City, with a value of 0.4959. The lowest value is 0.2432 in Yongjing County, Linxia Hui Autonomous Prefecture. The highest level of urban spatial integration in 2015 was in Weiyuan County, Dingxi City, with a value of 0.6290. The lowest value is 0.1507 in Guide County, Hainan Tibetan Autonomous Prefecture. The highest level of urban spatial integration in 2022 is in Weiyuan County, Dingxi City, with a value of 0.7498. The lowest value is in Yongjing County, Linxia Hui Autonomous Prefecture, with a value of 0.0934.

#### 3.2.6. The spatial pattern of comprehensive integration.

From an overall perspective, the spatial distribution of comprehensive integration presents a distinct “core-periphery” structure. The core areas are mainly concentrated in regions centered around Lanzhou and Xining. For instance, some districts and counties surrounding Lanzhou and areas near Xining are mostly classified as “High” or “Higher” grades in the figure. These regions exhibit a high level of comprehensive integration and serve as the core drivers of the urban agglomeration’s development. In contrast, the peripheral areas of the urban agglomeration, such as districts and counties far from the core cities, are mostly graded as “Low” or “Lower,” indicating a relatively low level of comprehensive integration. From a dynamic perspective, the scope of the core areas has shown a gradual expansion trend from 2012 to 2022. Although the overall integration level of the LXUA has improved, the development of peripheral areas is relatively slow, reflecting the persistent imbalance in development within the urban agglomeration.

### 3.3. Coupling driven detection and analysis of the comprehensive integration of LXUA

#### 3.3.1. Single factor detection.

According to the principle of the parameter optimal geographic detector model, the closer the q value is to 1, the stronger the explanatory power of the factor for the dependent variable, and vice versa. The contributions of various variable factors in the comprehensive integration of LXUA are shown in Table 2. The q-values of financial market activity, economic development quality, and urban-rural labor force ratio are the highest, at 0.922, 0.857, and 0.855, respectively, with explanatory power exceeding 85%, indicating that they are the main socio-economic factors affecting urban agglomeration integration. Secondly, the q-values of fiscal expenditure share, postal and telecommunications level, public education construction, and urbanization rate are 0.695, 0.611, 0.564, and 0.562, respectively. These variables demonstrate over 50% explanatory power, indicating that they are also important factors affecting the integration of urban agglomerations. The explanatory power of other variable factors for the dependent variable is also around 20%. In summary, all variable factors have strong explanatory power for the integration of the LXUA, among which financial market activity, economic development quality, urban-rural labor force ratio, fiscal expenditure share, postal and telecommunications level, public education construction, and urbanization rate are the main factors.

**Table 2:** The contribution level of a single factor to the comprehensive integration of urban agglomerations.

First level indicator	Secondary indicators	Symbol	*q*-value	Significance
Integration of economic development	Economic development quality	S1	0.857	0.000
	Share of fiscal expenditure	S2	0.695	0.001
	Activity level of financial markets	S3	0.922	0.000
Integration of ecological environment	Construction level of public green spaces	S4	0.445	0.455
	Atmospheric ecological protection	S5	0.484	0.037
	Water ecological protection	S6	0.262	0.596
Integration of infrastructure	Highway construction level	S7	0.215	0.488
	Postal and telecommunications level	S8	0.611	0.111
	Public education level	S9	0.564	0.158
Integration of public services	Educational services	S10	0.263	0.450
	Medical service	S11	0.206	0.143
	Social security services	S12	0.199	0.306
Integration of urban space	Urban rural integration	S13	0.855	0.000
	Urbanization rate	S14	0.562	0.031
	Urban-rural income gap	S15	0.348	0.342

#### 3.3.2. Dual factor coupling interaction detection.

**Fig 5:**
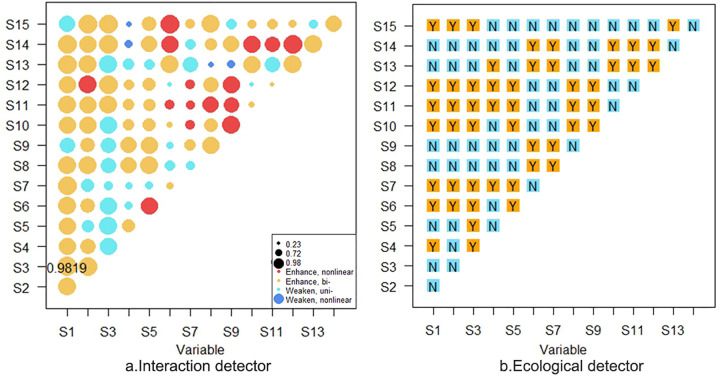
Dual factor interaction detection (a) and ecological detection (b).

In the factor coupling interaction detection, it was found that there is a significant interaction between nonlinear and bilinear enhancement effects among the factors affecting the comprehensive integration of urban agglomerations (as shown in Fig 5). For example, in addition to public education construction and urban-rural income gap, the interaction between economic development quality and other factors indicates a bilinear enhancement effect with a q-value greater than 0.9. This indicates that economic factors are almost independent of factors such as infrastructure, ecological environment, public services, and urban space, and there is no mutual influence. The impact on the comprehensive integration of urban agglomerations is a cumulative consequence of independent interpretations. In addition, there is a non-linear enhancement effect between water ecological protection and factors such as urbanization rate and urban-rural income gap, indicating that it has a significant impact on the comprehensive integration of urban agglomerations. The factors of public education construction and urbanization rate show non-linear enhancement effects with education services, medical services, and social security services, respectively. This indicates that the interaction between infrastructure factors and public service factors has a significantly greater explanatory power for the comprehensive integration of urban agglomerations than their independent effects, and there is a significant synergistic effect. At the same time, it indicates that the impact on the comprehensive integration of urban agglomerations is not simply cumulative, but exhibits unique interactive patterns.

## 4. Discussion

### 4.1. Framework for promoting the integrated development of urban agglomerations in underdeveloped regions

**Fig 6:**
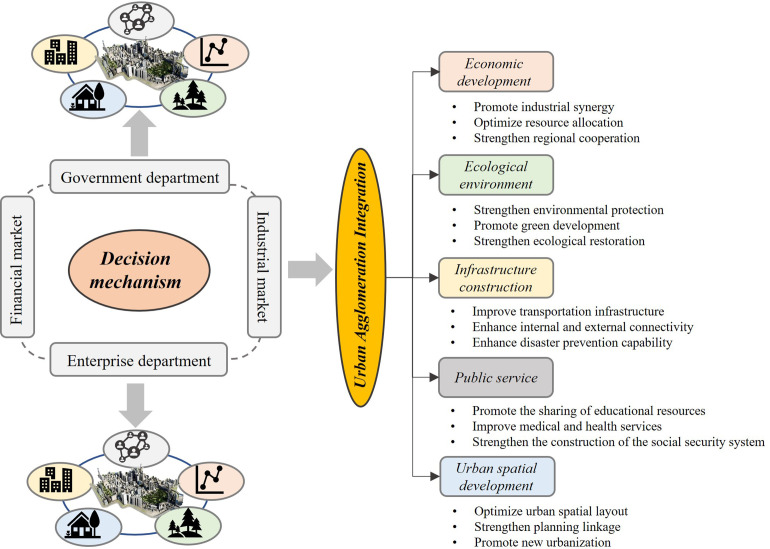
Strategic framework for promoting integrated development of urban agglomerations in underdeveloped regions.

The LXUA, as an important cross provincial urban agglomeration approved by the Chinese government for construction in the western region, is still in the process of cultivation. Currently, the overall level of integrated development in the LXUA is relatively low, especially the lowest level of economic integration. This indicates that the problem of insufficient and unbalanced economic development within the urban agglomeration is very prominent. In terms of space, there is a significant non-equilibrium feature, with high-value areas clustered around provincial capital cities. In response to the low level of integrated development in the LXUA. In response to the common problems faced by the integrated development of urban agglomerations in these underdeveloped areas, a framework that can be promoted is proposed (as shown in Fig 6).

Taking the LXUA as an example, the development measures proposed in combination with the strategic framework are as follows:

In terms of economic integration development. Firstly, we need to promote the coordinated development of industries in the LXUA. Strengthen the division of labor and cooperation within urban agglomerations, and form an industrial pattern of complementary advantages and differentiated development. Encourage and support enterprises within urban agglomerations to carry out technological innovation and industrial upgrading, and enhance overall competitiveness. Promote cooperation and co construction of industrial parks within urban agglomerations, share resources, and reduce production costs. Secondly, continuously improve the financial credit system and enhance the activity of the financial market. Thirdly, we need to balance and optimize the allocation of resources. Establish and improve the internal resource factor market of urban agglomerations, promote the free flow and optimized allocation of resources such as capital, technology, and talent. Strengthen financial cooperation within urban agglomerations, broaden financing channels, and reduce financing costs. Fourthly, actively promote regional economic cooperation, expand market space, and enhance the overall economic strength of urban agglomerations. Promote the integrated development of the economy within and around urban agglomerations, and build a new pattern of regional economic development.

In terms of integrated development of ecological environment. Firstly, further strengthen ecological environment protection, and especially for atmospheric ecological protection and urban ecological protection. Continuously establish and improve internal ecological environment protection mechanisms within urban agglomerations, and enhance environmental supervision and law enforcement efforts. Promote the coordinated governance of ecological environment within and around urban agglomerations, and jointly address environmental pollution and ecological damage issues. Secondly, efforts should be made to promote green development, encourage and support enterprises within urban agglomerations to adopt environmentally friendly technologies and processes, and reduce energy consumption and emissions. Promote the development of green industries within urban agglomerations and cultivate new economic growth points. Thirdly, we will strengthen ecological restoration and construction, implement key ecological restoration projects, and restore and improve the ecological environment quality within urban agglomerations. Strengthen the greening and beautification work within urban agglomerations, enhance the livability and ecological quality of cities.

In terms of integrated development of infrastructure. Firstly, accelerate the improvement of the transportation network, strengthen the construction of transportation infrastructure within urban agglomerations, and enhance the accessibility and convenience of the transportation network. Especially strengthening the resilience of transportation networks to enhance resistance to sudden public health emergencies and natural disasters.. Secondly, enhance energy and communication facilities, strengthen the construction of energy and communication infrastructure within urban agglomerations, and improve the level of energy supply and communication services. Promote the interconnection of energy and communication facilities within and around urban agglomerations, achieve resource sharing and optimized allocation. Thirdly, establish an inter-provincial emergency coordination mechanism, including the unified dispatching of medical resources, emergency transportation support, and an information sharing platform. Fourth, strengthen the construction of water conservancy and disaster prevention facilities, improve water conservancy infrastructure within the urban agglomeration, and enhance flood control, waterlogging drainage, and irrigation capacities. Comprehensively reinforce the construction of disaster prevention and response facilities in the urban agglomeration to improve the ability to cope with natural and man-made disasters.

In terms of integrated development of public services. Firstly, promote the sharing of educational resources, strengthen education cooperation and exchange within urban agglomerations, and promote the sharing of high-quality educational resources. Enhance the education level within urban agglomerations and cultivate high-quality talents. Secondly, improve medical and health services, strengthen cooperation and communication within urban agglomerations, and enhance the level of medical and health services. Promote the optimization and coordinated development of medical and health resources within and around urban agglomerations. Thirdly, strengthen the construction of the social security system: improve the internal social security system of urban agglomerations and enhance the level of social security. Promote the integration and coordinated management of social security systems within and around urban agglomerations.

In terms of integrated development of urban space. Firstly, optimize the urban spatial layout, formulate scientifically reasonable urban spatial planning, and optimize the urban spatial layout within the urban agglomeration. Promote the integrated development of urban spaces within and around urban agglomerations, and build a livable and business friendly urban system. Secondly, strengthen the connection between urban and rural planning, especially within urban agglomerations, to promote integrated urban-rural development. Promote the integrated development of urban and rural planning within and around urban agglomerations, and enhance the scientific and forward-looking nature of urban and rural planning. Thirdly, promote new urbanization and rural revitalization, accelerate the process of new urbanization within urban agglomerations, and improve the quality and level of urbanization. Promote the revitalization and development of rural areas within and around urban agglomerations, and promote coordinated urban-rural development.

### 4.2. Comparative analysis with existing research

This study comprehensively evaluates the integrated development level of the LXUA by constructing a “five-dimensional” evaluation system. In terms of the basic research framework, it continues the fundamental structure employed in existing studies on developed urban agglomerations. Consistent with findings in the literature regarding spatial differentiation in urban agglomerations, the results of this study indicate that areas with high levels of integrated development are primarily concentrated in provincial capitals, while peripheral counties and districts exhibit relatively lagging development.

Unlike most existing literature, which focuses on developed urban agglomerations such as the Yangtze River Delta, Pearl River Delta, and Chengdu-Chongqing regions, this research takes the LXUA, an underdeveloped urban agglomeration in Western China, as its case study. This fills a gap in the research on urban agglomeration integration in ecologically fragile and economically underdeveloped areas. While integration in developed urban agglomerations is predominantly driven by market forces and industrial collaboration, this study finds that integration in the LXUA relies more heavily on policy intervention and the construction of infrastructure connectivity. The research also reveals that the LXUA achieves the lowest level of integration in economic development, while public service and ecological environment integration are relatively higher. This stands in stark contrast to developed urban agglomerations, where economic integration typically takes the lead. This highlights the unique characteristic of underdeveloped regions: Economic lag but relatively low ecological pressure. This study proposes a “five-dimensional” regulatory framework of “Economy-Ecology-Infrastructure-Service-Space.” It emphasizes that for underdeveloped regions, priority should be given to addressing infrastructure shortcomings and the urban-rural income gap, rather than simply replicating the industrial upgrading paths of developed urban agglomerations. This strategy offers greater regional adaptability.

### 4.3. Research limitations and future prospects

This study takes the LXUA in the inland northwest of China as an example and constructs an evaluation index system for urban agglomeration integration using panel data from districts and counties spanning from 2012 to 2022. It analyzes the level of integration within the LXUA. The evaluation dimensions cover five aspects: economic development, ecological environment, infrastructure, public services, and urban spatial layout, making it relatively comprehensive overall. However, in the selection of indicators, on the one hand, due to factors such as statistical scales and data confidentiality, all selected indicators are at the county-level. This may lead to uncertainties in research results at a more refined scale. On the other hand, to balance the number of influencing factors across different dimensions, only a small number of indicators are chosen for each dimension. Although these indicators can generally reflect the integration level of urban agglomerations, in future evaluations, more indicators should be further selected in combination with actual conditions to enhance the comprehensiveness of the indicator system.

The data used in this study are all panel data. For urban integration development, the updating speed of these data may lag behind actual developments, resulting in a certain deviation between the analysis results and actual situations. In addition, the LXUA is located in the inland northwest of China, characterized by complex and diverse geographical conditions and harsh climatic conditions (such as drought and cold). These factors may have significant impacts on the integration development of the urban agglomeration. However, existing research may not have fully considered the specificity of these geographical and climatic factors. The development of urban agglomerations is influenced by national and local policies and institutional environments. Different policies and institutions may have varying effects in promoting integration development. Therefore, future research needs to further analyze the impact of policies and institutions on the integration development of the LXUA.

## 5. Conclusion

The article constructs an integrated measurement index system for the LXUA based on five aspects of integrated development: economic development, ecological environment, infrastructure, public services, and urban space. It analyzes the spatio-temporal characteristics of the integrated development level of the LXUA from 2012 to 2022. At the same time, using the parameter optimal geographic detector model, the coupling driving mechanism of urban agglomeration integration was revealed. The main conclusions drawn are as follows:

(1) There is a significant gap in the integration level dimension of the LXUA. Specifically, the level of integration of public services is the highest, followed by integration of ecological environment, while the level of integration of economic development is the lowest. This indicates that the problem of insufficient and unbalanced regional economic development in the LXUA is more prominent.
(2) The spatial differentiation characteristics of the integration level of LXUA are obvious. The high-value areas of integration are mainly concentrated in core cities such as Lanzhou and Xining, while underdeveloped counties lag behind in development. The spatial differences between urban areas have intensified, with significant differentiation between high-value and low value areas. The urban-rural gap and urbanization rate are key influencing factors.
(3) Single factor detection shows that financial market activity, economic development quality, urban-rural labor force ratio, fiscal expenditure share, postal and telecommunications level, public education construction, and urbanization rate are the main factors affecting the comprehensive integration level of urban agglomerations. The dual factor interaction detection indicates that economic factors have almost independent effects on infrastructure, ecological environment, public services, and urban space, and there is no mutual influence. However, the interaction between infrastructure factors and public service factors has a significantly greater explanatory power for the comprehensive integration of urban agglomerations than their independent effects, indicating a significant synergistic effect. Its impact on the comprehensive integration of urban agglomerations is not simply cumulative, but exhibits a unique interactive mode.
